# Accelerating caregivers’ HIV status disclosure to community-based lay social welfare volunteers in Tanzania

**DOI:** 10.1186/s12981-021-00332-4

**Published:** 2021-04-01

**Authors:** Amon Exavery, John Charles, Asheri Barankena, Erica Kuhlik, Godfrey Martin Mubyazi, Christina Kyaruzi, Tumainiel Mbwambo, Amal Ally, Remmy Mseya, Levina Kikoyo, Elizabeth Jere

**Affiliations:** 1 Pact, P.O. Box 6348, Dar es Salaam, Tanzania; 2 Pact, Inc., 1828 L St NW Suite 300, Washington, DC 20036 USA; 3 National Institute for Medical Research (NIMR), P.O Box 9653, Dar es Salaam, Tanzania

**Keywords:** HIV status, Disclosure, Caregivers of orphans and vulnerable children, Volunteers, Kizazi Kipya, Tanzania

## Abstract

**Background:**

HIV status disclosure facilitates access to HIV-related prevention and treatment services and increases opportunities for social support, HIV risk reduction with partners, and index testing for sexual partners or children. This study assessed the effect of a program model of community-based social welfare volunteers on HIV status disclosure among caregivers of orphans and vulnerable children (OVC).

**Methods:**

This was a longitudinal study, which was based on OVC caregivers who were beneficiaries of the USAID Kizazi Kipya project in Tanzania. They were enrolled (baseline) by community social welfare volunteers during 2017–2018, received services, and reassessed at midline in 2019. Caregivers who reported having been HIV tested, were asked to voluntarily report the status in order for the volunteers to establish and provide needed services. Those who reported their HIV status as negative or positive were grouped as “disclosed”, and those who knew their status but did not report it were documented as “undisclosed”. McNemar’s tests compared disclosure rates at baseline and midline. Multivariable analysis was conducted using generalized estimating equation (GEE).

**Results:**

The study analyzed 140,664 caregivers (72% female) from 81 district councils of Tanzania. Their mean age at enrollment was 47.4 years. Overall, 81.3% of the caregivers disclosed their HIV status to the project staff at baseline; this increased significantly to 96.1% at midline (p < 0.001). Disclosure at baseline varied significantly by sociodemographic characteristic (p < 0.05), with higher disclosure in females, among urban residents, and higher educated caregivers. However, the observed disclosure variations by sociodemographic characteristics at baseline disappeared at midline and disclosure reached around 96% across the characteristics (p > 0.05). In the multivariable analysis, caregivers’ likelihood of HIV status disclosure was nearly 6 times higher at midline than at baseline, when baseline characteristics were adjusted for (OR = 5.76, 95% CI 5.59–5.94, *p* < 0.001). There were 26,329 caregivers who did not disclose their HIV status at baseline (i.e., 0% diclosure rate at baseline), but 94.7% (n = 24,933) had disclosed by midline, and their disclosure trend was rapidly increasing as their duration of exposure to the project increased.

**Conclusions:**

This study detected an increased caregivers’ HIV status disclosure to the USAID Kizazi Kipya project staff by 14.8%, from 81.3% at baseline to 96.1% at midline within an average project exposure period of 1.4 years. The observed loss of sociodemographic differences in HIV status disclosure rate at midline implies that community-based interventions may be well-positioned to successfully address and eliminate sociodemographic barriers to service uptake and consequently improve services coverage and health outcomes.

## Background

HIV status disclosure facilitates entry into care and treatments programs. This is a key public health strategy for HIV prevention and control [1–3]. It has been observed that HIV status disclosure motivates sexual partners to seek HIV testing and change sexual practices and behaviours, which ultimately declines HIV transmission [3]. Disclosure is encouraged to reduce sexual risk behaviours, HIV transmission, and HIV-associated stigma [4, 5]. At an individual level, disclosure improves opportunities for social support, access to care and treatment services, as well as planning for the future [3].

Extant statistics of HIV status disclosure among sexual partners are diverse [6–8], and tends to show better rates in developed than developing countries [9]. Within Tanzania, disclosure rates vary by geography [10–12]. A recent study in Kilimanjaro found that HIV serostatus disclosure to partners was 66% [13], and ranged from 93.3% in Mwanza [14], to 28% in Morogoro [15]. One study in three government-owned health facilities in Pwani region of Tanzania, found that nurse-facilitated HIV status disclosure was only 39% among postpartum women who had not yet disclosed [16]. These variations highlight a need for further research to uncover factors encouraging HIV status disclosure, and interventions to address disclosure barriers.

Previous studies identified several factors associated with HIV status disclosure: stigma and discrimination [17–19], economic status [20, 21], literacy [22], gender [9], age [23–25], marital status [13, 23–25], being on ART, contraceptive use [13], knowledge of partner’s HIV status, membership in HIV/AIDS control associations [26], and many others [14, 15, 27–30]. In these studies, disclosure has mainly been that of: revealing one’s own HIV test results to a sexual partner, family members or friends [12, 13, 23, 31–36]; and HIV-infected children learning their status from their parents, guardians or caregivers [37–40].

Despite this evidence, little is known about HIV status disclosure to HIV prevention and treatment programs. Documented evidence of interventions enhancing HIV status disclosure is also rare. Some community-based HIV prevention and treatment programs promote voluntary disclosure of one’s HIV status in order to establish needed services. Therefore, undisclosed status, limits what the programs can achieve with respect to provision of HIV services targeted to the beneficiary’s unique HIV needs [41]. This highlights the need for interventions to support voluntary HIV status disclosure among individuals for health and social needs identification and timely provision of such services.

This study assessed the contribution of the USAID Kizazi Kipya project’s model of community-based service delivery using volunteers on HIV status disclosure rates among caregivers of orphans and vulnerable children (OVC) in Tanzania by comparing baseline and midline disclosure rates. The program, USAID Kizazi Kipya, scales-up service uptake among HIV infected or affected OVC and their caregivers. Therefore, this study was appropriate in this population because the population is already at high risk of and burdened by HIV. For example, early enrollee caregivers in this project had a self-reported HIV prevalence of 28.3% [42].

## Methods

### Data source

Data for this study are from a community–based project in Tanzania known as USAID Kizazi Kipya. The project (2016–2021) aims at scaling up the uptake of HIV, health, and social services by OVC and their caregivers. The data were collected by Lead Case Workers (LCWs) and Community Case Workers (CCWs) during beneficiary screening and enrollment using the project’s screening and enrollment, and Family and Child Asset Assessment (FCAA) tools. LCWs and CCWs are lay social welfare volunteers recruited by a government standard and trained in basic social welfare case management skills. Beneficiaries were enrolled into the USAID Kizazi Kipya project if their household met one or more of the enrollment criteria. The criteria refer to 14 household vulnerabilities related to HIV, one of which is whether one or more household members are HIV positive. The criteria are published [41].

### Services provided by the USAID Kizazi Kipya project to OVC caregivers

While the USAID Kizazi Kipya project serves both OVC and their caregivers, for the purpose of this study, only services provided to the caregivers are described (Table 1). The project works with LCWs and CCWs at the community level to provide services during household visits. Each household is visited at least once every calendar quarter. Services are provided in the areas of health and HIV, food and nutrition, psychosocial care and support, economic strengthening, education, and child protection. A list of categories of the services provided by the USAID Kizazi Kipya project is published [43]. While some of these services are provided directly, referrals are issued and tracked for the services that the project does not directly provide, such as HIV testing, HIV treatment, and many others. At enrollment, HIV status was enquired of each caregiver, observing their right to autonomy as well as respecting their privacy. This information informed the development of a care plan which responded to the holistic needs of each household member, including HIV needs of the caregiver.

**Table 1:** Direct and/or referral services provided by the USAID Kizazi Kipya project to OVC caregivers in Tanzania

Service domain	List of Services
Health	HIV testing and counseling
	HIV care and treatment
	ART adherence education
	HIV prevention education
	HIV disclosure support
	TB/HIV screening
	PMTCT services
	STI treatment services
	Opportunistic infections treatment (OIs)
	Home Based Care Services (HBC)
	Post Exposure Prophylaxis (PEP)
	Antenatal care services (ANC)
	Labor and delivery
	Postnatal services
	Family Planning (FP)
	Immunization
	Integrated management of childhood illness (IMCI)
	Early Childhood development /Care for Child Development
	Deworming
	Malaria prevention
	Diarrhea treatment
	Mental Health services
	Non-Communicable Diseases (NCDs)
Food and nutrition	Nutrition status assessment, counselling and support
	General food support
	Supplemental feeding services
	Therapeutic feeding services
Psychosocial care and support	Counselling
	Social participation
	Child welfare education
	Cultural and Spiritual support services
	Alcohol and Drug Abuse Support
Economic strengthening	Cash Transfers (TASAF)/Savings and Lending Support
	Income generating activity (IGA), small business/enterprise support
	Vocation skills support
	Agricultural and extension service support

### Study design and data collection tools

This study used a longitudinal design [44], whereby enrollment data (2017–2018) established the baseline, and, following one to 2½ years of service delivery, midline data was collected from the same beneficiaries in 2019. The FCAA tool which was used at both surveys, captured caregivers’ demographic information, household assets, sources of income, HIV status, food security, and use of and adherence to antiretroviral therapy (ART) for those who reported their HIV status as positive.

### Study area

Data for this study originate from 81 district councils in 25 regions of Tanzania (44% of district councils and 81% of regions in the country) where the USAID Kizazi Kipya project had implemented screening and enrollment activities in 2017–2018.

### Study population

The current study is based on a cohort of 140,664 OVC caregivers who were enrolled in the USAID Kizazi Kipya project during 2017–2018 and reported that they have tested for HIV and know their HIV status. Caregivers who had not tested for HIV at enrollment, or exited the project for any reason (e.g. lost to follow-up (LTFU), died or moved away) before 2019, or not interviewed for the midline survey were not included in this study. The study followed the caregivers through project service delivery between 2017 and 2019, and their midline assessment in 2019. Therefore, each caregiver included in the current study had two measurements of HIV status disclosure: one at the baseline and the other at the midline. A caregiver is defined by the USAID Kizazi Kipya project as a guardian who has the greatest responsibility for the daily care and rearing of one or more OVC in a household. A caregiver is not necessarily a biological parent of the OVC.

### Variables

Caregivers’ voluntary disclosure of their own HIV status to the USAID Kizazi Kipya project’s volunteers (i.e. LCWs or CCWs) was the outcome variable for the current study. Caregivers who reported that they have been tested for HIV, were subsequently asked to voluntarily report their test results in order for the volunteer to establish and provide needed services. Caregivers who reported their HIV results as negative or positive were grouped together and referred to as ‘disclosed’, and those who knew their status but did not report it to the volunteer were documented as ‘undisclosed’.

Pre and post intervention timings of HIV status disclosure was used in this study as the explanatory variable of interest. This fell into two categories: (1) baseline disclosure, i.e. HIV status disclosed at enrollment prior to USAID Kizazi Kipya service delivery, and (2) midline disclosure, i.e. HIV status disclosed to the USAID Kizazi Kipya project staff after the caregiver had received USAID Kizazi Kipya services for up to 2½ years depending on their enrollment date.

Other explanatory variables included in this study were caregiver sex, age, education, marital status, household level of hunger, type of residence (rural or urban), and physical or mental disability. Rural residence included all those living in district councils, whereas those living in townships, municipal or city councils were classified as urban residents. Household level of hunger was measured using the Household Hunger Scale (HHS) [45] and had three categories: (1) little to no hunger (food secure), (2) moderate hunger, and (3) severe hunger.

### Data analysis

All statistical analyses were performed using Stata 14.0 statistical software. Distributional features of the caregivers were obtained through one-way tabulations. For both baseline and midline surveys, the association between HIV status disclosure and each of the independent variables was tested using the Chi-Square (χ^2^) test because all variables were categorical. Comparison between baseline and midline disclosure rates was made using McNemar’s test because baseline disclosure observations of the same caregivers were paired to their midline counterparts [46–48]. Disclosure rates in each of the individual categories of the independent variables at midline and baseline were compared using immediate form of two-sample test of proportions [49].

Multivariable analysis of factors associated with HIV status disclosure among the caregivers was conducted using generalized estimating equation (GEE) with logit link function, binomial distribution family and an exchangeable correlation structure. Since HIV status disclosure was measured twice (i.e., at baseline and at midline) for each caregiver, their disclosure observations were assumed to be correlated. Therefore, this made the GEE the most appropriate model because it addresses within-subject correlations and both time-dependent and time-independent covariates [50–55]. The model took the following form:

$${\mathrm{Y}}_{\mathrm{it}}={\upbeta }_{0}+\sum_{\mathrm{j}=1}^{\mathrm{J}}{\upbeta }_{1\mathrm{j}}{\mathrm{X}}_{\mathrm{itj}}+{\upbeta }_{2}\mathrm{t}+\dots +{\mathrm{CORR}}_{\mathrm{it}}+{\upvarepsilon }_{\mathrm{it}}$$

where Y_it_ are HIV status disclosure observations for caregiver i at time t, β_0_ is the intercept, X_ijt_ is the independent variable j for caregiver i at time t, β_1j_ is the regression coefficient for independent variable j, J is the number of independent variables, t is time (t_0_ = baseline, and t_1_ = midline), β_2_ is the regression coefficient for time, CORR_it_ is the working correlation structure (exchangeable in this case), and ε_it_ is the ‘error’ term for caregiver i at time t. HIV status disclosure was measured at two time points: at baseline (t_0_) where caregivers were being enrolled into the project and had not received any services from the USAID Kizazi Kipya project; and at the midline (t_1_) where all caregivers had received program services. In the model specification, coefficients were exponentiated using Stata’s ‘*eform’* option to obtain adjusted odds ratios (ORs) and their corresponding 95% confidence intervals (CIs). Statistical inferences were made at a significance level of 5%, whereby factors corresponding with *p*–values ≤ 0.05 were considered statistically significant in the prediction of HIV status disclosure.

## Results

### Baseline profile of respondents

As shown in Table 2, the current study is based on 140,664 caregivers of OVC, 72.2% of whom were female. At enrollment (the baseline), they were aged at least 18 years and their mean age was 47.4 years (standard deviation [SD] = 14.2). About half (49.4%) of the caregivers were married or living together with their spouses; and 76.1% had some primary education.

**Table 2:** Baseline socio-demographic profile of respondents

Variable	Number of Caregivers (n)	Percent (%)
ALL	140,664	100.0
Sex		
Female	101,557	72.2
Male	39,107	27.8
Age (years)		
18–29	11,292	8.0
30–39	34,184	24.3
40–49	40,522	28.8
50–59	24,405	17.4
60 +	30,261	21.5
Mean = 47.4, SD = 14.2	–	–
Marital status		
Married or living together	69,421	49.4
Divorced or separated	17,386	12.4
Never been married	9941	7.1
Widow/widower	43,916	31.2
Education		
Never attended	28,704	20.4
Primary	107,092	76.1
Secondary +	4868	3.5
Place of residence		
Rural	76,548	54.4
Urban	64,116	45.6
Household Hunger Scale		
Little to no hunger	37,257	26.5
Moderate hunger	91,147	64.8
Severe hunger	12,260	8.7
Caregiver mentally or physically disabled?		
No	135,799	96.5
Yes	4865	3.5

Slightly more than a half (54.4%) resided in rural areas; and 3.5% were mentally or physically disabled. With respect to food security, the majority (64.8%) were in households with moderate hunger, and 8.7% in severe hunger households (Table 2).

**Fig. 1:**
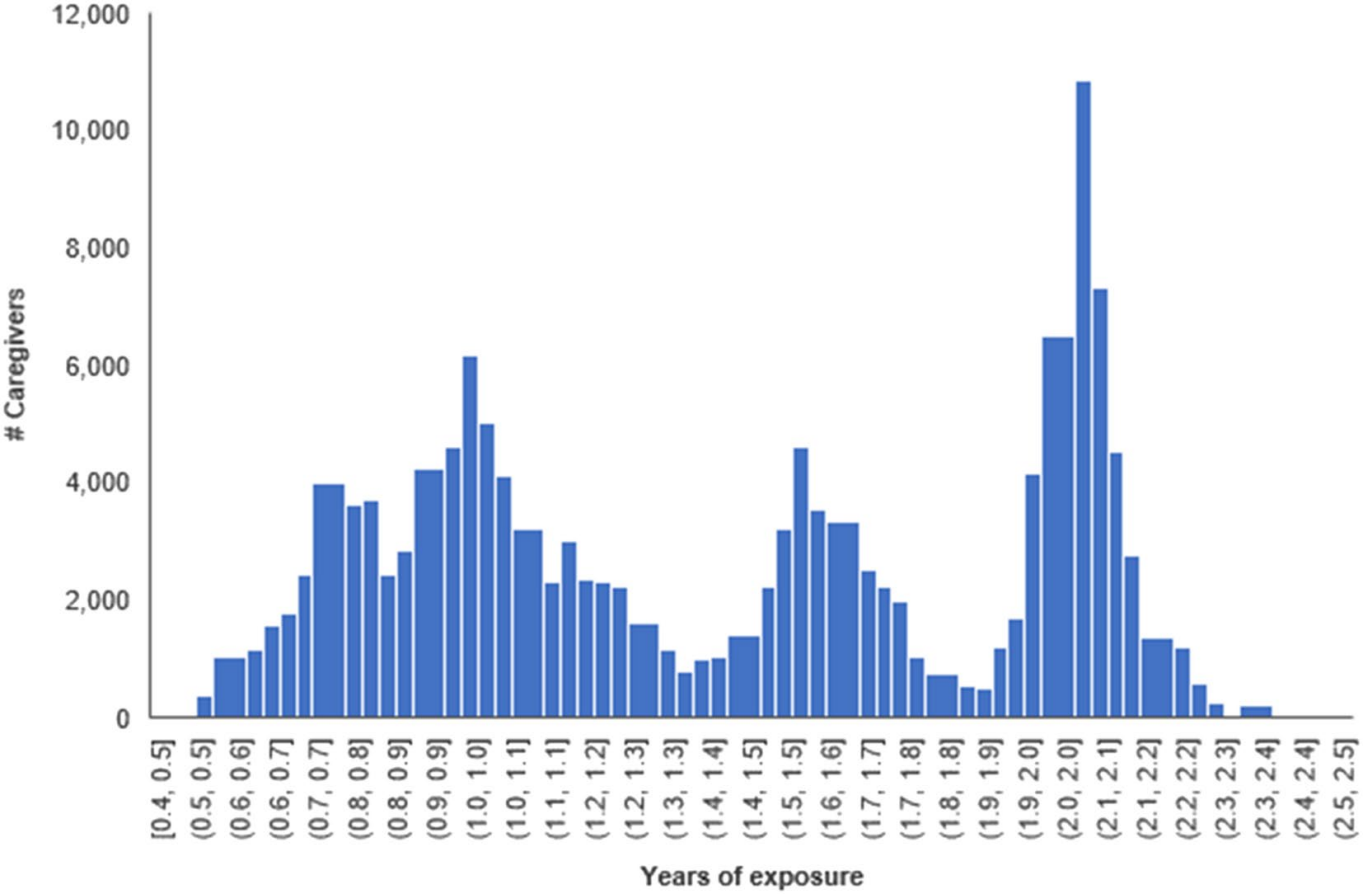
Caregivers’ duration of exposure to the USAID Kizazi Kipya project from baseline (enrollment) to midline (n = 140,664)

Between baseline and midline, the caregivers had received services from the USAID Kizazi Kipya project for different durations depending on their enrollment dates. Earlier enrollees received services for a longer duration than those who enrolled later. From baseline (enrollment) to midline, the mean duration of exposure to the project was 1.4 years, ranging from 0.4 to 2.5 years (SD = 0.5) (Fig. 1).

### HIV status disclosure rates b etween baseline and midline

**Fig. 2:**
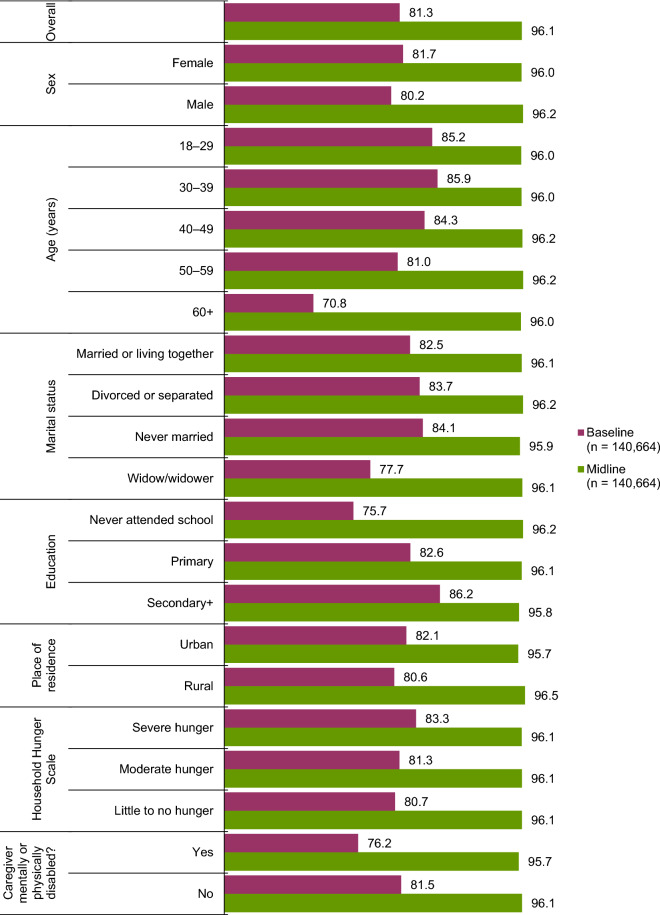
Rates (%) of HIV status disclosure at baseline and at midline by baseline characteristics among 140,664 caregivers of OVC in Tanzania

As presented in Fig. 2, overall, caregivers’ HIV status disclosure to the USAID Kizazi Kipya project staff was 81.3% at baseline and 96.1% by midline, (an increase of 14.8%, p < 0.001). Similarly, McNemar’s comparison of the disclosure rates at the two time points indicated that, the likelihood of disclosure was six times higher at midline than at baseline (odds ratio [OR] = 6.1, 95% confidence interval [CI] 5.9–6.3). Caregivers who disclosed their HIV status were subsequently linked to appropriate services per their status, and those who did not were encouraged to do so as soon as they are comfortable. Referral services provided by the project are published elsewhere [43]. Ultimately, all the caregivers, regardless of their HIV status, were linked to other program services depending on established needs.

### HIV status disclosure rates between baseline and midline, by baseline characteristics

As seen in Fig. 2, caregivers’ disclosure of their HIV status to the USAID Kizazi Kipya project staff at baseline varied significantly by their baseline characteristics: female caregivers were more likely to disclose than their male counterparts (p < 0.001). After age 39, disclosure declined consistently to the lowest level of 70.8% among the oldest caregivers aged 60 years and above (p < 0.001). Disclosure was highest among caregivers who were single or never married and lowest among those who were widowed (p < 0.001). With respect to education, the rate of disclosure increased consistently with education, and pinnacled to 86.2% among caregivers with secondary education or more (p < 0.001). Disclosure was better in urban than in rural areas (p < 0.001). The disclosure rate was lower among caregivers who were mentally or physically disabled than those who were not (p < 0.001). Finally, the rate of HIV status disclosure among the caregivers increased steadily as the level of household hunger rose (p < 0.001).

At the midline, apart from place of residence—whereby rural caregivers were more likely to disclose than their urban counterparts (p < 0.001)—the variations in rates of HIV status disclosure by the caregivers’ baseline characteristics were no longer present (p > 0.050), and the rate was around 96.0% across the characteristics.

### Midline disclosure rates by duration of exposure to the USAID Kizazi Kipya project

There were 26,329 caregivers who did not disclose their HIV status to the program staff at baseline. Of these, 94.7% (n = 24,933) disclosed at midline, and 10.2% (n = 2,675) of them were HIV positive and subsequently linked to appropriate HIV services. Those who were HIV negative received HIV prevention knowledge and where they can access more information about HIV and AIDS. Again, all caregivers, regardless of their HIV status, were linked to other program services depending on established needs.

**Fig. 3:**
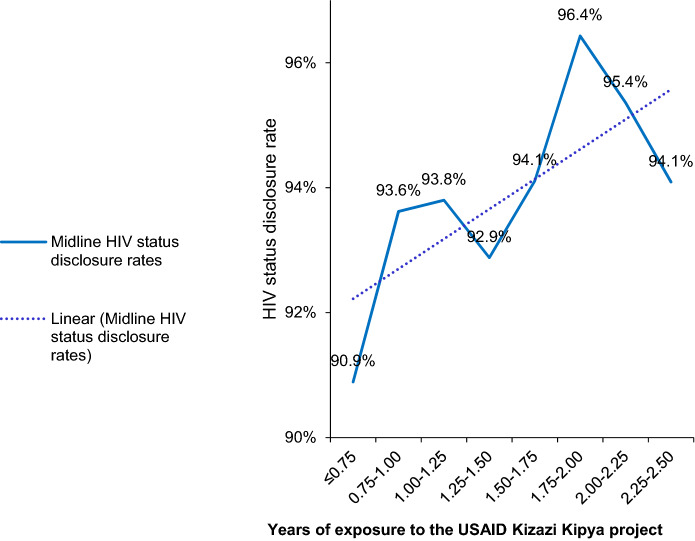
Midline HIV status disclosure rates among 26,329 caregivers who had not disclosed their HIV status at baseline by duration of exposure to the USAID Kizazi Kipya project

As shown in Fig. 3, overall, HIV status disclosure in this group had an increasing trend with an increasing duration of exposure to the program, from 90.9% among caregivers who had been in the program for 9 months or less to 94.1% among caregivers who had been in the program for more than 2¼ years. The highest disclosure rate was 96.4% among caregivers who had been in the program between 1¾ and 2 years.

**Fig. 4:**
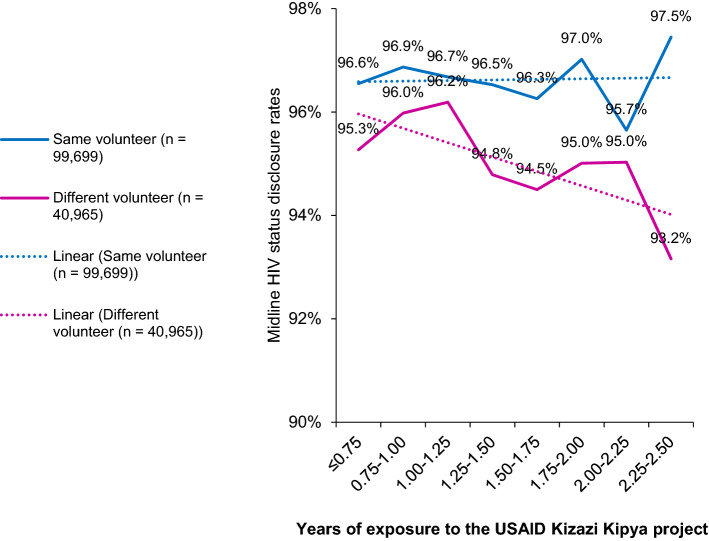
Midline HIV status disclosure rates (stratified by whether the volunteer changed) by duration of exposure to the USAID Kizazi Kipya project

In Fig. 4, all the 140,664 caregivers’ midline HIV status disclosure rates by the duration of exposure to the USAID Kizazi Kipya project were stratified by whether there was a change of the volunteer. Results showed that, the disclosure trend was slightly increasing as the duration of exposure to the project increased if the volunteer remained the same but decreasing if the volunteer was different.

**Fig. 5:**
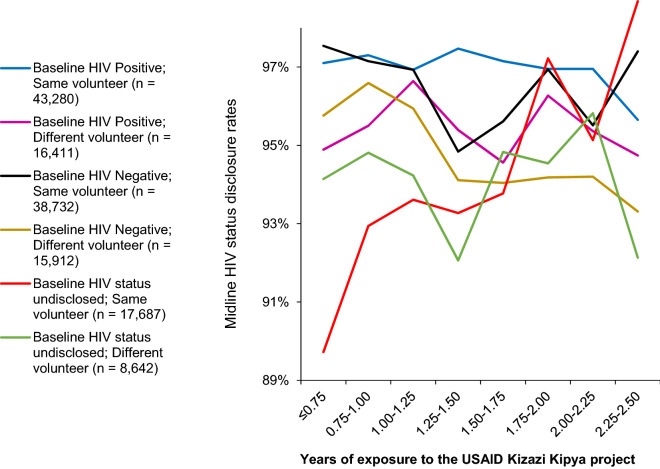
Caregivers’ midline HIV status disclosure rates stratified by baseline HIV status and whether the volunteer changed by duration of exposure to the USAID Kizazi Kipya project

Further stratifications of the midline disclosure rates were made by considering baseline HIV status (positive, negative, and undisclosed) and whether the volunteer remained the same from baseline to midline (Fig. 5). Results revealed that the disclosure trend was rapidly increasing over time for caregivers who had not disclosed their HIV status at baseline and had the same volunteer (n = 17,687) (from 89.7% for caregivers who had been in the project for 9 months or less to 98.7% for those who had been in the project for more than 2¼ years). The rest of the disclosure trends for different groups of the caregivers were not significantly changing over time, except those who were HIV negative at baseline and had a different volunteer (n = 15,912) whose disclosure trend was significantly declining (from 95.8 to 93.3% among those who had been in the project for 9 months or less and more than 2¼ years, respectively).

### Increase in disclosure rates from baseline to midline by baseline characteristics

**Fig. 6:**
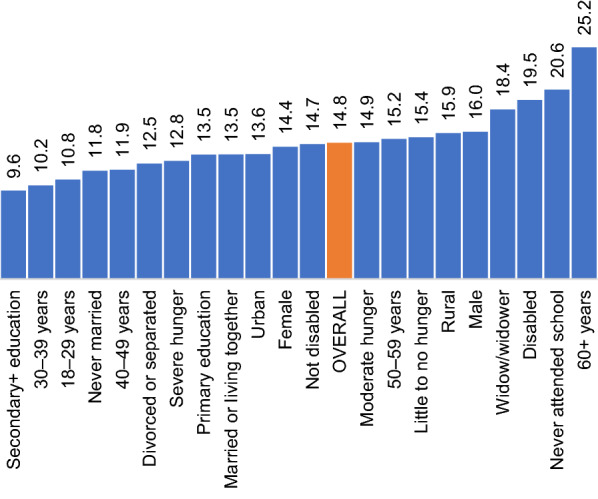
Increase (%) in HIV status disclosure rates from baseline to midline by baseline characteristics among 140,664 caregivers in Tanzania

Figure 6 is derived from Fig. 2 and shows the amount of change in disclosure rates between baseline and midline across different caregivers’ baseline characteristics. The project effect on disclosure (as measured at midline) was largest where disclosure was lowest at baseline, ranging from 9.6% among those who had secondary + education to 25.2% among those who were aged 60 years or more, with an average disclosure gain of 14.8% (Fig. 6).

### Results of multivariable analysis

**Fig. 7:**
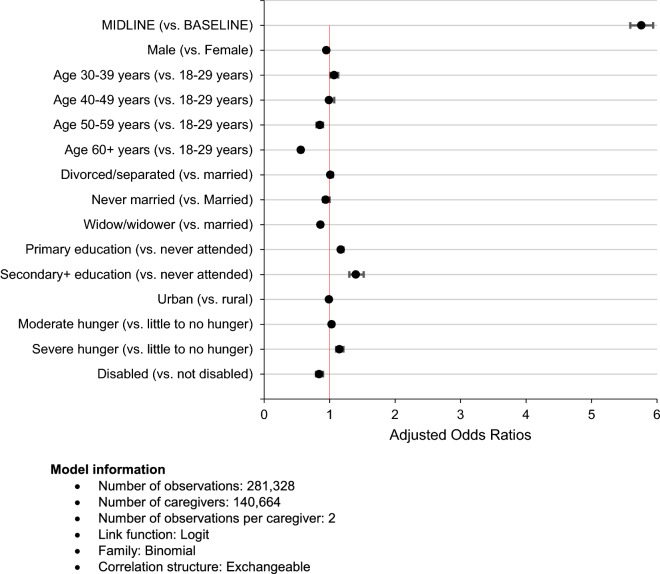
Multivariable generalized estimating equation (GEE) of factors associated with HIV status disclosure among OVC caregivers in Tanzania

Figure 7 shows multivariable GEE model of factors associated with HIV status disclosure among the caregivers. Adjusted odds ratios (ORs) and their corresponding 95% confidence intervals (CIs) are presented. Factors are adjusted for one another in the model, and the subsequent interpretations embeds this fact.

The analysis uncovered that caregivers’ likelihood of HIV status disclosure was nearly 6 times higher at midline than at baseline, when the baseline characteristics were adjusted for (OR = 5.76, 95% CI 5.59–5.94, *p* < 0.001).

In addition, with all factors adjusted for one another, male caregivers were 5% less likely to disclose their HIV status than their female counterparts (OR = 0.95, 95% CI 0.92–0.98, p < 0.001). Caregivers in the age group 30–39 years were 7% more likely to disclose their HIV status than the youngest age 18–29 years (OR = 1.07, 95% CI 1.01–1.13, p = 0.017). Subsequent age groups were each less likely to disclose their HIV status than the youngest age group, with significance only for age groups 50–59 years (OR = 0.85, 95% CI 0.80–0.90, p < 0.001), and 60 + years (OR = 0.56, 95% CI 0.53–0.59, p < 0.001). Widowed caregivers were 14% less likely compared to those who were married or living together with their spouses to disclose their HIV status (OR = 0.86, 95% CI 0.84–0.89, p < 0.001). With respect to education, both primary (OR = 1.17, 95% CI 1.14–1.21, p < 0.001) and secondary or more education (OR = 1.40, 95% CI 1.30–1.52, p < 0.001) levels were more likely to improve HIV status disclosure than no education.

Furthermore, the likelihood of HIV status disclosure increased with increasing levels of household hunger, from 3% among caregivers in moderate hunger (OR = 1.03, 95% CI 1.00–1.06, p = 0.049) to 15% among those in severe hunger households (OR = 1.15, 95% CI 1.10–1.21, p < 0.001). Finally, caregivers who were physically or mentally disabled were 16% less likely to disclose their HIV status than those without the disabilities (OR = 0.84, 95% CI 0.79–0.90, p < 0.001).

## Discussion

This study assessed the contribution of the USAID Kizazi Kipya project’s model of community-based service delivery through social welfare volunteers on HIV status disclosure rates among 140,664 caregivers of OVC in Tanzania at baseline and at midline. Results showed an increase of 14.8% in caregivers’ HIV status disclosure to the program staff from 81.3% at baseline to 96.1% at midline in an average exposure period of 1.4 years. In the multivariable analysis, HIV status disclosure was about six times more likely at midline than at baseline, when baseline characteristics of the caregivers were adjusted for. Note that at the midline, all the caregivers had received some services from the USAID Kizazi Kipya project, including disclosure support for those who had not disclosed their HIV status at the baseline. This means that the project successfully improved caregivers’ HIV status disclosure, and consequently expanded service coverage.

Out of the caregivers who did not disclose their HIV status at baseline (i.e., 0% disclosure rate at baseline), 94.7% disclosed at midline, and their disclosure trend was increasing as their duration of exposure to the program increased. This increase was very rapid if the volunteer remained the same. The USAID Kizazi Kipya project intervention contributed to this achievement, especially through the disclosure support services provided to the caregivers who had not disclosed their HIV status at baseline. This means that the project was successful in encouraging HIV status disclosure among the caregivers, and consequently expanded coverage of its HIV services, thus contributing to the United Nations’ sustainable development goal 3 of universal health coverage [56]. The community volunteers’ facilitated HIV status disclosure rate observed in this study was much higher than the health provider facilitated rate of 39% estimated in a similar study among postpartum women who had not yet disclosed their status in three health facilities in Tanzania [16].

On the other hand, if the volunteer changed over the course of service delivery, the disclosure trends became horizontal or declining over time in different groups of the caregivers, particularly those who disclosed their HIV status at baseline as HIV negative. Although the change of the volunteer was due to causes beyond the project control (e.g. drop out, death etc.), the finding cemented the importance of trust [57] in HIV status disclosure which takes time to build. Therefore, introducing a new volunteer to the caregivers implied that the volunteer-caregiver relationship building had to start afresh, hence resulting in declining disclosure rates: some of the caregivers who disclosed at baseline didn’t disclose at midline, mainly because the volunteer changed. This highlights the need for community-based volunteer-driven programs to devise mechanisms to minimize dropout of volunteers because it is pernicious, and this may not be limited to HIV status disclosure. Further research is needed to explore factors affecting volunteers’ satisfaction and retention, and suggest possible ways to address their drop out in community-based programs. Also, research should explore the extent to which trust, safety and comfortability are ensured during the process of handing over caregivers and other beneficiaries of community-based programs to new volunteers.

In addition, this study uncovered several other factors with significant association with HIV status disclosure. With respect to sex, multivariable analysis showed that male caregivers were less likely to disclose than their female counterparts. This was also observed in one review study [9]. Previous studies have reported poor health seeking behaviour among men than women [58, 59], which is why they may also be less likely to disclose their HIV status. The current study demonstrates that the intervention was effective in bridging the disclosure gap between men and women, thus a need to continue disclosure support interventions, with additional support for men. As similarly observed in other studies [13, 23–25], HIV status disclosure in the current study was less likely among widowed than married caregivers. It is expected that caregivers in marital unions are more likely to disclose to their spouses because they are in contact with each other for improved communication and trust in each other than those who are widowed [13]. Therefore, since widowed caregivers may have difficulties in disclosing their HIV status, possibly due to lack of social support [60] and or stigma and discrimination, targeted disclosure support interventions are important. In the current study, the observed differences in disclosure rates by marital status at baseline disappeared at midline, hence revealing effectiveness of the USAID Kizazi Kipya project in addressing gender-related barriers to HIV status disclosure. Further research should explore specific gender-related factors that influence HIV status disclosure.

Disclosure by age pinnacled among those who were aged 30–39 years, after which the likelihood to disclose started to decline in the subsequent age groups 40–49, 50–59, and 60 + years than the youngest caregivers in the age group 18–29 years. These observations are similar to others in Kenya [23] and Nigeria in [24]. Further research is required to explore why this was the case.

The likelihood of HIV status disclosure increased with increasing education. This is consistent with other studies [22, 32]. This suggests that as better education improves self-esteem and confidence [61], communication skills improves as well [32] and consequently contributing to improved HIV status disclosure. Therefore, while formal education attainment should continue to be emphasized and expanded for universal coverage for longer term gain, interventions to enhance disclosure such as the USAID Kizazi Kipya are necessary especially for those who are unable to go back to school.

Surprisingly, the likelihood of HIV status disclosure increased with increasing levels of household hunger. Unfortunately, we did not clearly understand how this relationship came into existence. We thus recommend further research to explore and explain the underlying pathways for this observation. In the descriptive analysis, the differences in disclosure rates by household level of hunger were significant at baseline but vanished at the midline following the USAID Kizazi Kipya intervention. This suggests that the project successfully addressed hunger-related barriers to HIV status disclosure, and the current efforts should be sustained to ultimately achieve total disclosure.

Finally, disclosure was less likely among caregivers who were mentally or physically disabled than those without the disabilities. Some studies have shown that disability connotes stigma [62, 63], a situation which has been shown that it increases fears of disclosure (9). Therefore, disabled people should be targeted with additional disclosure support. With the disclosure support provided by the USAID Kizazi Kipya project, disclosure rates at the midline were similar among caregivers with and without disability.

## Limitations

While the USAID Kizazi Kipya project invests heavily towards enhancing HIV status disclosure and service uptake among beneficiaries, an optimum quantification of its effect may be difficult to realize because the inherent design of the project did not comprise a control group.

## Conclusions

Lay social welfare volunteers provide continuous and frequent household support for a wide range of needs, such as economic strengthening, parenting, and violence prevention and response. Through the volunteers known as LCWs and CCWs, the USAID Kizazi Kipya project in Tanzania contributed to the increased caregivers’ HIV status disclosure by 14.8%, accelerating from 81.3% at baseline to 96.1% at midline within an average exposure period of 1.4 years. Of the caregivers who did not disclose their HIV status at baseline (i.e., 0% disclosure rate at baseline), 94.7% had disclosed by midline and their disclosure trends increased with increasing duration of exposure to the program, and the increase was very rapid if the volunteer remained the same from baseline to midline.

The observed differences in disclosure rates by baseline characteristics were clearly larger at baseline, but had disappeared by midline, suggesting that the USAID Kizazi Kipya project greatly contributed to this achievement through its regular services provided through the community-based volunteers (i.e., LCWs and CCWs). The increase in disclosure rates between baseline and midline across all the caregivers’ baseline characteristics were higher among caregivers who were less likely to disclose at baseline and vice versa. This suggested that the intervention gain was equitable among the caregivers, whereby the more needy experienced more gain in terms of HIV status disclosure, and ultimately all became similar at midline. This implies that community-based interventions are better-positioned to successfully address and possibly eliminate sociodemographic and contextual barriers to HIV status disclosure and possibly uptake of services and consequently improve health outcomes.

## Data Availability

The datasets analyzed during the current study are owned by Pact Tanzania and are not publicly available due to confidentiality restrictions. Therefore, access to the data can be provided by Pact Tanzania only.

## Abbreviations

AIDS: Acquired immunodeficiency syndrome
ART: Antiretroviral therapy
CCWs: Community Case Workers
CHF: Community health fund
CI: Confidence interval
FCAA: Family and child asset assessment
HIV: Human immunodeficiency virus
HHS: Household hunger scale
IHI: Ifakara Health Institute
LCWs: Lead Case Workers
LHIV: Living with HIV
LTFU: Loss/lost to follow-up
MRCC: Medical Research Coordinating Committee
NIMR: National Institute for Medical Research
OR: Adjusted odds ratio
OVC: Orphans and vulnerable children
PLHIV: People living with HIV
SD: Standard deviation
UNAIDS: Joint United Nations Programme on HIV and AIDS
USAID: United States Agency for International Development

## References

[1] The Lancet HIV (2018). HIV status disclosure in a digital age. Lancet HIV.

[2] Tang W, Cao B, Liu C, Pan S, Zhang Y, Ong J (2017). HIV serostatus disclosure from partners before sex: results from an online survey of Chinese men who have sex with men. Lancet.

[3] WHO. HIV Status Disclosure to Sexual Partners: Rates, Barriers and Outcomes for Women . Geneva, Switzerland: WHO; 2004. https://apps.who.int/iris/bitstream/handle/10665/42717/9241590734_summary.pdf;jsessionid=13CCE59FA4F342FFC01C415CB44913AA?sequence=2. Accessed 7 May 2020.

[4] King R, Katuntu D, Lifshay J, Packel L, Batamwita R, Nakayiwa S (2008). Processes and outcomes of HIV serostatus disclosure to sexual partners among people living with HIV in Uganda. AIDS Behav.

[5] Miller AN, Rubin DL (2007). Factors leading to self-disclosure of a positive HIV diagnosis in Nairobi, Kenya: people living with HIV/AIDS in the Sub-Sahara. Qual Health Res.

[6] Shacham E, Small E, Onen N, Stamm K, Overton ET (2012). Serostatus disclosure among adults with HIV in the era of HIV therapy. AIDS Patient Care STDS.

[7] Cissé M, Diop S, Abadie A, Henry E, Bernier A, Fugon L (2016). Factors associated with HIV voluntary disclosure to one’s steady sexual partner in mali: results from a community-based study. J Biosoc Sci.

[8] Conserve DF, King G, Dévieux JG, Jean-Gilles M, Malow R (2014). Determinants of HIV serostatus disclosure to sexual partner among HIV-positive alcohol users in Haiti. AIDS Behav.

[9] Obermeyer CM, Baijal P, Pegurri E (2011). Facilitating HIV disclosure across diverse settings: a review. Am J Public Health.

[10] Antelman G, Smith Fawzi MC, Mbwambo J, Msamanga GI, Hunter DJ, Fawzi WW (2001). Predictors of HIV-1 serostatus disclosure: a prospective study among HIV-infected pregnant women in Dar es Salaam. Tanzania AIDS.

[11] Kilewo C, Massawe A, Lyamuya E, Semali I, Kalokola F, Urassa E (2001). HIV counseling and testing of pregnant women in sub-Saharan Africa: experiences from a study on prevention of mother-to-child HIV-1 transmission in Dar es Salaam Tanzania. J Acquir Immune Defic Syndr.

[12] Kiula ES, Damian DJ, Msuya SE (2013). Predictors of HIV serostatus disclosure to partners among HIV-positive pregnant women in Morogoro Tanzania. BMC Public Health.

[13] Damian DJ, Ngahatilwa D, Fadhili H, Mkiza JG, Mahande MJ, Ngocho JS (2019). Factors associated with HIV status disclosure to partners and its outcomes among HIV-positive women attending Care and Treatment Clinics at Kilimanjaro region, Tanzania. PLoS ONE.

[14] Yonah G, Fredrick F, Leyna G (2014). HIV serostatus disclosure among people living with HIV/AIDS in Mwanza Tanzania. AIDS Res Ther.

[15] Hallberg D, Kimario TD, Mtuya C, Msuya M, Björling G (2019). Factors affecting HIV disclosure among partners in Morongo, Tanzania. Int J Afr Nurs Sci.

[16] Geubbels E, Williams A, Ramaiya A, Tancredi D, Young S, Chantry C. HIV status disclosure among postpartum women in rural Tanzania: predictors, experiences and uptake of a nurse-facilitated disclosure intervention. AIDS Care. 2018. 10.1080/09540121.2018.1428724. Accessed 3 Aug 2020. 10.1080/09540121.2018.1428724 PMC6250839 29363340

[17] Przybyla SM, Golin CE, Widman L, Grodensky CA, Earp JA, Suchindran C (2013). Serostatus disclosure to sexual partners among people living with HIV: examining the roles of partner characteristics and stigma. AIDS Care.

[18] Hosseinzadeh H, Hossain SZ, Bazargan-Hejazi S (2012). Perceived stigma and social risk of HIV testing and disclosure among Iranian-Australians living in the Sydney metropolitan area. Sex Health.

[19] Dageid W, Govender K, Gordon SF (2012). Masculinity and HIV disclosure among heterosexual South African men: implications for HIV/AIDS intervention. Cult Health Sex.

[20] Longinetti E, Santacatterina M, El-Khatib Z (2014). Gender perspective of risk factors associated with disclosure of HIV status, a cross-sectional study in Soweto South Africa. PLoS ONE.

[21] Antelman G, Smith Fawzi MC, Kaaya S, Mbwambo J, Msamanga GI, Hunter DJ (2001). Predictors of HIV-1 serostatus disclosure: a prospective study among HIV-infected pregnant women in Dar es Salaam Tanzania. AIDS.

[22] Taraphdar P, Dasgupta A, Saha B (2007). Disclosure among people living with HIV/AIDS. Indian J Commun Med.

[23] Trinh TT, Yatich N, Ngomoa R, McGrath CJ, Richardson BA, Sakr SR (2016). Partner disclosure and early CD4 Response among HIV-infected adults initiating antiretroviral treatment in Nairobi Kenya. PLoS ONE.

[24] Amoran OE (2012). Predictors of disclosure of sero-status to sexual partners among people living with HIV/AIDS in Ogun State Nigeria. Niger J Clin Pract.

[25] Agbor IE, Etokidem AJ, Ugwa EA. Factors Responsible for Disclosure of HIV Seropositivity among Residents of Cross River State, Nigeria. In: Indian journal of community medicine : official publication of Indian Association of Preventive & Social Medicine. 2017. 10.4103/ijcm.IJCM_313_15 PMC5561689 28852275

[26] Alema HB, Yalew WA, Beyene MB, Woldu MG (2015). HIV positive status disclosure and associated factors among HIV Positive Adults in Axum Health Facilities, Tigray, Northern Ethiopia. Sci J Public Health.

[27] Gultie T, Genet M, Sebsibie G (2015). Disclosure of HIV-positive status to sexual partner and associated factors among ART users in Mekelle Hospital. HIV/AIDS Res Palliat Care.

[28] Hayes-Larson E, Hirsch-Moverman Y, Saito S, Frederix K, Pitt B, Maama BL (2017). Prevalence, patterns, and correlates of HIV disclosure among TB-HIV patients initiating antiretroviral therapy in Lesotho. AIDS Care.

[29] Ramlagan S, Matseke G, Rodriguez VJ, Jones DL, Peltzer K, Ruiter RAC (2018). Determinants of disclosure and non-disclosure of HIV-positive status, by pregnant women in rural South Africa. SAHARA J..

[30] Whembolua G-L, Conserve DF, Thomas K, Tshiswaka DI, Handler L (2019). HIV serostatus disclosure in the Democratic Republic of the Congo: a systematic review. AIDS Care.

[31] Atuyambe LM, Ssegujja E, Ssali S, Tumwine C, Nekesa N, Nannungi A (2014). HIV/AIDS status disclosure increases support, behavioural change and HIV prevention in the long term: a case for an Urban Clinic, Kampala, Uganda. BMC Health Serv Res.

[32] Kadowa I, Nuwaha F (2009). Factors influencing disclosure of HIV positive status in Mityana district of Uganda. Afr Health Sci.

[33] Kiarie JN, Kreiss JK, Richardson BA, John-Stewart GC (2003). Compliance with antiretroviral regimens to prevent perinatal HIV-1 transmission in Kenya. AIDS.

[34] Maman S, Mbwambo JK, Hogan NM, Weiss E, Kilonzo GP, Sweat MD (2003). High rates and positive outcomes of HIV-serostatus disclosure to sexual partners: reasons for cautious optimism from a voluntary counseling and testing clinic in Dar es Salaam. Tanzania AIDS Behav.

[35] Medley A, Garcia-Moreno C, McGill S, Maman S (2004). Rates, barriers and outcomes of HIV serostatus disclosure among women in developing countries: implications for prevention of mother-to-child transmission programmes. Bull World Health Organ.

[36] Walcott MM, Hatcher AM, Kwena Z, Turan JM (2013). Facilitating HIV status disclosure for pregnant women and partners in rural Kenya: a qualitative study. BMC Public Health.

[37] Sariah A, Rugemalila J, Somba M, Minja A, Makuchilo M, Tarimo E (2016). “Experiences with disclosure of HIV-positive status to the infected child”: Perspectives of healthcare providers in Dar es Salaam, Tanzania. BMC Public Health.

[38] Vaz LME, Maman S, Eng E, Barbarin OA, Tshikandu T, Behets F (2011). Patterns of Disclosure of HIV- Status to Infected Children in a Sub-Saharan African Setting. J Dev Behav Pediatr.

[39] Vreeman RC, Gramelspacher AM, Gisore PO, Scanlon ML, Nyandiko WM (2013). Disclosure of HIV status to children in resource-limited settings: a systematic review. J Int AIDS Soc.

[40] Pinzón-Iregui MC, Beck-Sagué CM, Malow RM (2013). Disclosure of Their HIV status to infected children: a review of the literature. J Trop Pediatr.

[41] Exavery A, Charles J, Kuhlik E, Barankena A, Koler A, Kikoyo L (2020). Understanding the association between caregiver sex and HIV infection among orphans and vulnerable children in Tanzania: learning from the USAID Kizazi Kipya project. BMC Health Serv Res.

[42] Exavery A, Charles J, Kuhlik E, Barankena A, Mubyazi GM, Kikoyo L (2020). Relationship between food insufficiency and HIV infection among caregivers of orphans and vulnerable children in Tanzania. HIV/AIDS Res Palliat Care.

[43] Bajaria S, Abdul R, Exavery A, Minja E, Charles J, Mtenga S (2020). Programmatic determinants of successful referral to health and social services for orphans and vulnerable children: A longitudinal study in Tanzania. PLoS ONE..

[44] Pearce N (2012). Classification of epidemiological study designs. Int J Epidemiol.

[45] Ballard T, Coates J, Swindale A, Deitchler M. Household Hunger Scale: Indicator Definition and Measurement Guide . Washington, DC: Food and Nutrition Technical Assistance II Project, FHI 360; 2011 p. 23. https://www.fantaproject.org/sites/default/files/resources/HHS-Indicator-Guide-Aug2011.pdf

[46] Caronni A, Sciumè L (2017). Is my patient actually getting better? Application of the McNemar test for demonstrating the change at a single subject level. Disabil Rehabil.

[47] Fagerland MW, Lydersen S, Laake P (2013). The McNemar test for binary matched-pairs data: mid-p and asymptotic are better than exact conditional. BMC Med Res Methodol.

[48] Westfall PH, Troendle JF, Pennello G (2010). Multiple McNemar Tests. Biometrics.

[49] Kim H-Y (2016). Statistical notes for clinical researchers: sample size calculation. 2 Comparison of two independent proportions. Restor Dent Endod..

[50] Diggle P, Liang K-Y, Zeger SL (1994). Analysis of longitudinal data.

[51] Liang K-Y, Zeger SL (1986). Longitudinal data analysis using generalized linear models. Biometrika.

[52] Twisk JWR (2003). Applied Longitudinal Data Analysis for Epidemiology: A Practical Guide.

[53] Zeger SL, Liang K-Y, Albert PS (1988). Models for longitudinal data: a generalized estimating equation approach. Biometrics.

[54] Zeger SL, Liang KY (1992). An overview of methods for the analysis of longitudinal data. Stat Med.

[55] Zeger SL, Liang K-Y (1986). Longitudinal data analysis for discrete and continuous outcomes. Biometrics.

[56] United Nations. #Envision2030 Goal 3: Good Health and Well-being . 2015. https://www.un.org/development/desa/disabilities/envision2030-goal3.html. Accessed 15 May 2020.

[57] Ssali SN, Atuyambe L, Tumwine C, Segujja E, Nekesa N, Nannungi A (2010). Reasons for Disclosure of HIV Status by People Living with HIV/AIDS and in HIV Care in Uganda: An Exploratory Study. AIDS Patient Care STDS.

[58] Abaerei AA, Ncayiyana J, Levin J (2017). Health-care utilization and associated factors in Gauteng province. South Africa Glob Health Action.

[59] Staveteig S, Wang S, Head SK, Bradley SEK, Nybro E. Demographic patterns of HIV testing uptake in sub-Saharan Africa . Calverton, Maryland, USA: ICF International; 2013 [cited 2019 Apr 11]. Report No.: DHS Comparative Reports No. 30. https://dhsprogram.com/publications/publication-CR30-Comparative-Reports.cfm. . Accessed 11 Apr 2020.

[60] Dessalegn NG, Hailemichael RG, Shewa-amare A, Sawleshwarkar S, Lodebo B, Amberbir A (2019). HIV Disclosure: HIV-positive status disclosure to sexual partners among individuals receiving HIV care in Addis Ababa, Ethiopia. PLoS ONE.

[61] Dominic B. ‘Women’s Education a Tool of Social Transformation’-A Historical Study Based on Kerala Society. 2011;2(10):7.

[62] Corrigan PW (2014). The stigma of disease and disability: Understanding causes and overcoming injustices.

[63] Chanvilay T, Yoshida Y, Reyer JA, Hamajima N (2015). Factors associated with access to antiretroviral therapy among people living with HIV in Vientiane capital, LAO PDR. Nagoya J Med Sci.

